# The inequity of the Swiss health care system financing from a federal state perspective

**DOI:** 10.1186/1475-9276-13-17

**Published:** 2014-02-14

**Authors:** Luca Crivelli, Paola Salari

**Affiliations:** 1 Department of Economics, Università della Svizzera Italiana (USI), Via Buffi 13, 6900 Lugano, Switzerland; 2 Department of Business and Social Sciences, University of Applied Sciences of Southern Switzerland (SUPSI), Palazzo E, Manno CH-6928, Switzerland

**Keywords:** Switzerland, Equity, Health care system financing, Fiscal federalism

## Abstract

**Introduction:**

Previous studies have shown that Swiss health-care financing is particularly regressive. However, as it has been emphasized in the 2011 OECD Review of the Swiss Health System, the inter cantonal variations of income-related inequities are still broadly unexplored. The present paper aims to fill this gap by analyzing the differences in the level of equity of health-care system financing across cantons and its evolution over time using household data.

**Methods:**

Following the methodology proposed by Wagstaff et al. (JHE 11:361–387, 1992) we use the Kakwani index as a summary measure of regressivity and we compute it for each canton and for each of the sources that have a role in financing the health care system. We graphed concentration curves and performed relative dominance tests, which utilize the full distribution of expenditures.

The microdata come from the Swiss Household Income and Expenditure Survey (SHIES) based on a sample of the Swiss population (about 3500 households per year), for the years 1998 - 2005.

**Results:**

The empirical evidence confirms that the health-care financing in Switzerland has remained regressive since the major reform of 1996 and shows that the variations in equity across cantons are quite significant: the difference between the most and the least regressive canton is about the same as between two extremely different financing systems like the US and Sweden. There is no evidence, instead, of a clear evolution over time of regressivity.

**Conclusions:**

The significant variation in equity across cantons can be explained by fiscal federalism and the related autonomy in the design of tax and social policies. In particular, the results highlight that earmarked subsidies, the policy adopted to smooth the regressivity of the premiums, appear to be not enough; in the practice of federal states the combination of allowances with mandatory community-rated health insurance premiums might lead to a modest outcome in terms of equity.

## Introduction

The idea that health-care services should be paid for according to the ability to pay rather than according to the actual use of the health-care system finds its roots in the egalitarian concept of social justice and is generally adopted in Switzerland, as it is in most of the OECD countries. It is common in the economic literature to indicate this idea using the concept of *equity in financing*[1]. This principle implies not only a form of solidarity between the sick and the healthy, which is implicit in any health insurance system, but also solidarity between rich and poor. In this context, two measures of equity exist. The first is “horizontal equity”, which claims *equal treatments for equals*; i.e., that people with the same income have to contribute the same amount of money to the total expenditures. The second measure is “vertical equity”, which states that people with different income must contribute appropriate amounts to the total expenditure.

All of the empirical studies on this topic measure the equity of a financing source in terms of progressivity; i.e., the extent to which higher-income people pay more as a proportion of their income than lower- income people.

When the new Health Insurance Act (HIA) came into force in 1996, many things changed in the Swiss health-care system. The main objectives of the act were to guarantee universal coverage, to establish a fair competition among health insurers and to equip the political system with better cost containment tools. Moreover, it also increased the importance of the financing equity aim: one of the objectives of the reform was to provide monetary assistance to low-income households in order to increase equity of financing. In the federal draft bill issued in the year 1991 (and approved some years later after many amendments as HIA) we can read that: *The main priority of the project is undoubtedly the strengthening of solidarity. The current law provides for individual premiums to be paid without taking into account the economic situation of the people insured (p.67).* In order to make this payment affordable for all citizens, the new law replaced the historically grown system of general state transfers to health insurance with a better endowed and more equitable system of allowances targeted to low-income households.

At present the health insurance in Switzerland is mandatory for every citizen, and it is based on community-rated premiums. The confederation jointly with cantons assigns a budget cap for earmarked subsidies targeting low-income households. Cantons have a great autonomy in deciding the distribution of subsidies; despite their role of mitigating vertical inequity, the heterogeneity of cantonal subsidy policies leads to a different treatment of similar households across cantons (horizontal inequity). As subsidies vary with cantons, people with the same income living in different cantons may pay a different amount of money for health care.

Some studies have demonstrated that the general level of health system financing is regressive in Switzerland, both before and after the reform [2,3]. This means that lower-income people pay more as a proportion of their income than higher-income people.

Nevertheless, these studies consider only the whole of Switzerland and no research has monitored the situation at the cantonal level. Due to the Swiss federal setting, each canton differs in the economic strategy it has adopted to finance the health care system; this leads to different levels of equity among them. The OECD Review of the Swiss Health System [4] considers the development of an information system able to monitor this inter-cantonal variation as one of the Swiss policy challenges for the future.

This paper aims to fill this information gap by analyzing the level of equity in the financing of the health-care system in the Swiss cantons. In this study we have used the methodology suggested by Wagstaff et al. [5] to compute the regressivity level of each canton through the Kakwani index [6].

The paper is organized as follows. The next section presents a review of the most relevant literature in this field. We have collected and summarized similar studies conducted for other countries, as well as for all of Switzerland. The subsequent sections include: a brief explanation of Swiss health system financing; the description of the dataset and the methodology used; the discussion of the results obtained, some lessons for a federal state, and the limitations of the study. Finally, the last section offers some conclusions.

## Literature review

This work finds its place among the studies about the equity of health-care system financing.

Table 1 includes the main papers that have been published on this topic. Some of them have focused on a single country – namely Australia, Iran, Finland, Italy, the Netherlands, Malaysia, Palestine, Ireland, Tanzania, Ghana, Sweden, and Switzerland. Two papers present an international comparison, based on 13 Asian countries and 13 OECD countries, respectively.

**Table 1:** Main papers about equity in the health-care financing system

**Country**	**Author(s) and year**
Italy	Paci and Wagstaff (1993) [7]
Australia	Lairson et al. (1995) [8]
The Netherlands	Wagstaff and Van Doorslaer (1997) [9]
Finland	Klavus (1998 and 2001) [10,11]
Sweden	Gerdtham and Sundberg (1998) [12]
Malaysia	Yu et al. (2008) [13]
Switzerland	Bilger (2008) [3]
Palestine	Abu-Zaineh et al. (2008) [14]
Ireland	Smith (2010) [15]
Tanzania	Mtei et al. (2012) [16]
Iran	Alireza (2011) [17]
Ghana	Akazili et al. (2011) [18]
13 OECD countries	Wagstaff et al. (1999) [2]
10 OECD countries	Wagstaff et al. (1992) [5]
13 Asian countries	O’Donnell et al. (2008) [19]

Smith [15], Klavus [10], Akazili et al. [18], and Abu-Zaineh et al. [14] analyzed the inequity in financing through the Kakwani index as well as through a “disaggregated approach”; the latter provides summary measures over specific income groups, by using the dominance test to assess the level of progressivity for different deciles of income distribution.

Another different method consists of separating the redistributive effect into three parts: vertical, horizontal, and reranking effect. The works by Lairson et al. [8] for Australia and Gerdtham and Sundberg [12] for Sweden concentrate on this approach. Bilger [3] adopted the same methodology in the case of Switzerland.

Many studies have analyzed the equity of health system financing at the national level, while very few studies have explored this issue at the subnational level. Abu-Zaineh et al. [14] and Alireza [17] extended the analysis by computing the Kakwani index for two different regions of a given country: the former included in the study the two regions of the Occupied Palestinian territory (i.e. the West Bank and the Gaza Strip) and the latter applied the Kakwani index to urban and rural areas of Iran.

As far as Switzerland is concerned, the issue of the health-care system financing has been explored by Wagstaff et al. [2,5] and Bilger [3]. All three papers provide evidence in favor of highly regressive financing.

Wagstaff et al. [5] presented an international comparison of health-care financing across 10 countries. The score of the Kakwani index for Switzerland was based on 1982 data and was significantly negative (-0.117). Moreover, the Swiss healthcare financing was the second most regressive, after the U.S. (Kakwani index of -0.145).

In 1999, the same authors, along with some others, updated their previous paper with more recent data. In line with their previous work, the analysis for Switzerland shows that the new Kakwani index, based on the data of 1992, is -0.1402. In ten years the inequity level of financing grew even worse.

Bilger [3] provided another important contribution to the analysis of financing equity in Switzerland. For his analysis, he used the Swiss Household Income and Expenditure Survey (SHIES) of 1998, which contains data from 9295 Swiss households. He found evidence that health system financing in post-reform Switzerland remains very regressive. In particular, the paper concludes that the reform failed to reduce vertical inequity.

Starting from this strand of literature, the current paper aims to go a step further. The goal is to analyze the differences in the regressivity of the financing system at a sub-national level. Apart from the two previously noted exceptions (focusing on two regions of the same country), this is the first attempt to provide a systematic analysis of financing equity at the level of the single entities of a federal state (in this case, Switzerland at the cantonal level). Switzerland is an ideal country in which to add this new piece of evidence, as it can be set in a context of fiscal federalism, where the cantons have large freedom to decide on the financing sources for health care and in the design of the tax system [20].

## Swiss health system financing

### General framework

The Swiss health care system is based on a mixed private-social health insurance, and it is financed through several sources (in brackets the 2009 shares on the total health care costs): public expenditure (28%), mandatory health insurance (29%), general social insurances (6%), complementary health insurance (9%), co-payments and out-of-pocket (27%), other private (1%) expenses.

#### Public expenditure

Federal, cantonal and local authorities contribute to the health care system mainly in two forms:

• Direct subsidy of specific services (inpatient hospital care, nursing homes, home care), mostly paid by cantons, and payment of public health interventions (like prevention and health promotion).

• Indirect financing through earmarked subsidies for lower-income people who cannot afford mandatory health insurance, funded jointly by the Confederation and the cantons, and other means-tested allowances for specific target groups.

All these activities are financed through tax revenues: they include both direct and indirect taxes for the Confederation, mostly direct taxes for cantons and municipalities. In particular direct taxes consist of income taxes for the Confederation and both income and property taxes for cantons and municipalities.

#### Mandatory health insurance (MHI)

The health insurance in Switzerland, mandatory since the reform of 1996, consists of a monthly community rated premium that does not depend on the level of income. However, different premiums are set for two age-classes: lower premiums for children (age <19) and higher for adults. Moreover, insurers are free to grant a discount for students between 19 and 25. Health insurance is offered through a variable number of private nonprofit sickness funds (ranging from 118 in 1998, to 85 in 2005 and to the current number of 66 in 2012). It covers a comprehensive benefit basket fixed at the federal level. Coverage starts once the yearly deductible (minimum of 300 CHF, about 330 US$, in the standard contract) has been reached and includes a 10 percent copayment (up to a maximum amount of 700 CHF per year). People can opt for higher deductibles (up to 2500 CHF) and get in exchange a premium discount. Premiums are set at the regional level by each health insurer. Some cantons are considered as a single premium community, whereas others (including urban and rural areas) are divided in two or three regions (the total number of premium communities declined between 1998 and 2005 from 78 to 43). As a consequence, premiums significantly differ across regions and cantons, reflecting the huge differences in health care expenditures. Significant variation exists also across health insurers [21], due in particular to a still ineffective risk adjustment mechanism [22]. Horizontal equity across geographic communities is not guaranteed at all, since individuals with the same income pay significantly more or less depending on what premium region they are resident in. Moreover, not even horizontal equity within the same premium region can be completely achieved, due to competition in insurance market, switching decisions by consumers, different deductibles and the option to sign managed care contracts.

As mentioned before, for people earning an income under a certain threshold, the Confederation and the cantons jointly fund earmarked subsidies that cover part or the total of the monthly premium (full coverage concerns in particular retired and disabled people receiving means-tested benefits).

#### General social insurances

This is Bismarckian insurance system designed to cover through in cash benefits other (non-health) risks like longevity, disability and accident. In some circumstances it provides also benefits through in kind health care services. It covers, in particular, spending for rehabilitation in case of disability and the health care costs in case of professional and non-professional accidents of employed persons.

#### Complementary health insurance

People can choose to pay a risk-rated premium (for individual contracts) or a community-rated premium (for collective contracts) to have a voluntary supplementary private health insurance. It covers some health-care services or inpatient hospital amenities that are not included in the compulsory benefit basket.

#### Out-of-pocket

The out-of-pocket expenses include the co-participation to costs (namely the deductible and the 10% co-payment) as well as all other health care expenses not covered in the general contract (e.g. nursing homes fees, dental care, OTC drugs, etc.).

Some of the financing strategy rules are decided at the federal level. However, Swiss federalism allows cantons to make their own decision regarding the exact design of the financing policy they want to adopt.

The cantonal scope of action can take several forms. Firstly, a different mixture of the financing sources chosen by each canton^a^ determines a different level of regressivity: cantons that rely more on the MHI than on public expenditure are more likely to be regressive than those that choose to finance the system more through general taxation. Secondly, cantonal autonomy due to fiscal federalism allows significant leeway in deciding the subsidies policy for the worse-off [23-25]. Cantons are allowed to make their own decisions regarding the eligibility criteria for receiving benefits, which creates heterogeneity in their distribution and which leads to differences in the level of regressivity among cantons. Thirdly, each canton has significant leeway in the definition of the tax rates for income and property taxes, whereas each municipality decides every year the percentage of the cantonal tax liability to be paid as local taxes.

According to cantons’ choices, different equity (or inequity) levels are thus determined.

### Evidence of heterogeneity at the cantonal level

Due to data availability problem, in the analysis that follows we are forced to rely only on the first three funding sources in the list, i.e. public financing, MHI and general social insurances. The part of financing that we are considering is called *socialized* health expenditure (SHE), since it reflects collective spending for the universally accessible basket of health care benefits. It accounts for approximately 60 - 65% of the total financing in Switzerland.

Hence, in our analysis, we will decompose SHE in four sources: *federal taxes* (including direct tax and VAT), *cantonal and municipal taxes* (henceforth we will refer to this second group of tax simply as *cantonal tax*), *mandatory health insurance* (computed as net premium, i.e. the difference between the privately paid premium and the earmarked subsidies), and *general social insurances*^b^.

The first column in Table 2 considers the amount of the SHE for each canton and for Switzerland as a whole in absolute per capita values for 2005. The second column shows the incidence of SHE on average disposable income.

**Table 2:** Socialized health expenditure – absolute value (2005) and share of each financing source (average 1998–2005)

	**SHE per capita (CHF)**	**SHE as a share of average disposable income**	**Sources of financing**			
			**Federal taxes**	**Cantonal and municipal taxes**	**General social insurances**	**Mandatory health insurance (net premiums)**
*Zurich (ZH)*	4198	6.66%	10%	29%	13%	48%
*Bern (BE)*	4297	8.52%	11%	30%	13%	46%
*Lucerne (LU)*	3461	7.19%	13%	25%	16%	46%
*Uri (UR)*	3379	6.85%	13%	26%	16%	45%
*Schwyz (SZ)*	3581	6.14%	13%	25%	15%	47%
*Obwald (OW)*	3155	7.40%	14%	27%	16%	43%
*Nidwald (NW)*	3385	6.21%	13%	27%	16%	44%
*Glarus (GL)*	3541	8.01%	12%	30%	14%	44%
*Zug (ZG)*	3885	5.15%	12%	30%	14%	44%
*Fribourg (FR)*	3876	8.42%	12%	28%	14%	46%
*Solothurn (SO)*	4002	7.78%	11%	26%	14%	49%
*Basel City (BS)*	5854	9.31%	8%	39%	9%	44%
*Basel Land (BL)*	4197	7.60%	11%	27%	13%	49%
*Schaffausen (SH)*	4070	9.06%	11%	31%	13%	45%
*Appenzell O. Rhodes (AR)*	3505	6.33%	13%	27%	16%	44%
*Appenzell I. Rhodes (AI)*	2988	6.81%	16%	20%	19%	45%
*St. Gall (SG)*	3465	6.76%	13%	24%	15%	48%
*Grisons (GR)*	3837	7.18%	12%	29%	14%	45%
*Argovia (AG)*	3575	6.60%	13%	20%	15%	52%
*Thurgovia (TG)*	3204	7.10%	14%	19%	16%	51%
*Ticino (TI)*	4852	9.66%	10%	29%	12%	49%
*Vaud (VD)*	4858	9.01%	9%	30%	11%	50%
*Valais (VS)*	3732	8.32%	12%	30%	14%	44%
*Neuchâtel (NE)*	5017	10.44%	9%	33%	11%	47%
*Geneva (GE)*	6578	10.10%	7%	43%	8%	42%
*Jura (JU)*	4381	10.43%	10%	30%	12%	48%
***Switzerland (CH)***	**4243**	**7.85%**	**11%**	**29%**	**13%**	**47%**

The last four columns of Table 2 represent the proportion of the expenditure financed by each source. We computed these weights as an average for the considered period (1998 - 2005).

The first evidence from the table relates to the absolute level of expenditure which differs greatly across cantons. With respect to the Swiss average of 4243 CHF (registered in 2005, the latest year considered in the empirical analysis), canton Geneva and Basel City present the highest value of expenditures, with 6578 and 5854 CHF, respectively. Cantons Appenzell Inner Rhodes and Obwald register the smallest expenditure values (with 2988 CHF and 3155 CHF, respectively). As far as the incidence is concerned, it ranges from 5.15% of disposable income in the rich and low SHE canton of Zug to almost the double in Geneva, Jura and Neuchâtel. Whereas in Geneva it is the high level of SHE that determines the heavy burden for households, in Jura (where SHE is aligned with Swiss average) the high incidence is rather driven by a low average income.

However, the differences do not only concern the total amount of expenditure: another evident difference between cantons is the proportion of each of the single financing sources.

The table also shows that, in cantons where the total expenditure is higher, the share of the cantonal part tends to be also higher. This is because, where health care costs (and consequently premiums) are higher, many households cannot afford anymore the cost of mandatory health insurance, which means that cantons are forced to spend more in terms of subsidies for the worse-off.

The values in the table show that the most important part is financed by health insurers. This accounts, on average, for 47 percent of the SHE. The second source derives from the cantonal taxes that cover 29 percent of the SHE, followed by the social insurances and federal taxes that account for 13 percent and 11 percent, respectively.

Finally, it is worth noting the magnitude of the differences among cantons. There are two cantons for which the part financed by cantonal taxes is considerably larger (Geneva - 43 percent; Basel City - 39 percent), whereas in Appenzell Inner Rhodes the share financed by cantonal taxes corresponds to only 20 percent.

## Dataset

The micro dataset is the Swiss Household Income and Expenditure Survey (SHIES), which allows computation of household income at a very detailed level, including all the taxes and social contributions paid, as well as the health expenditures (premiums and, only to some extent, out-of-pocket expenditures) and indemnities related to health (subsidies from the state and reimbursements from the insurance companies).

This survey is available from 1998 to 2005 (excluding 1999) and is based on a sample of the Swiss population (approximately 3500 households observed for each of the years between 2000 and 2005, and 9295 observations for 1998).

The SHIES does not guarantee that the households sample is representative at the level of all cantons for each year, but only at the level of seven macro-regions, each of which groups cantons together according to their geographical position^c^. As the focus of this work is to control for the differences due to the federal setting, we were interested in maintaining the cantonal dimension. For this reason, we decided to merge the dataset of each year to obtain a representative sample at the cantonal level. This has been possible because different people were interviewed each year for each canton. In this way we were able to control for the cantonal differences, but we lost the information about the variation over time for each canton.

Ultimately, this study concentrates on the between-variation for each canton and on the within-variation only for the whole Switzerland and the seven macro-regions. There are some differences in the questionnaires, especially for the year 1998 with respect to the other years. Only in the wave of interviews taken in 1998, were people asked to report all the medical expenses (greater than 150 CHF) they had during the whole year preceding the month of the survey and not only all the medical expenses they had during the month of the interview (as it is in the other waves of the survey). Moreover, only for the year 1998 responders had to register the reimbursements received by the insurance companies during the whole year; hence there was a correspondence between the expenditures and their reimbursements. The collection of this data makes it possible to compute the out-of-pocket expenditures for each households and, consequently, to have a robust proxy for the yearly out-of-pocket financing at least for the year 1998.

## Methods: Kakwani index, concentration curves and dominance test

In order to measure the regressivity of each financing source, we used the procedure proposed by Wagstaff et al. [2], (see also [26] for the empirical implementation). We started by computing the Kakwani index for each financing source and then we computed a total Kakwani index aggregating these results with a weighted average, using as weights the proportion of each financing source with respect to the total financing. This procedure has been applied to 23 cantons^d^, to the seven macro-regions, and to Switzerland as a whole.

This index is computed as the difference between the concentration index of each financing source and the Gini index calculated on the (equivalent disposable) household income before considering any health-related expenditures (we refer to this as *pre-health* income). If the two curves coincide, the difference between the CI and the Gini index is zero and the financing source can be considered as proportional to income.

The formula is as follows:

$$K_{\mathrm{ip}}=CI_{\mathrm{ip}}–\mathrm{Gini}\_\mathrm{pr}e_{i\;}\;\text{Range:}\;\left[-2\text{:}+1\right]$$

where *i* indicates the geographical unit of analysis and *p* the different financing sources available in the dataset.

*CI*_*ip*_ is the concentration index of each financing source *p*. CI is twice the area between the concentration curve of the source *p* and the 45-degree line. It indicates whether the variable of interest is more concentrated among the poor (the concentration curve lies above the equality line and the index has a negative value) or among the rich (the curve lies below the equality line and the index has a positive value).

*Gini_pre*_*i*_ is the Gini index for the *pre-health* income.

Since the Swiss fiscal system does not rely on earmarked taxes for health care, we simply imputed to the amount of taxes paid by each household the share of total health expenditure financed by that tax. We did this for federal tax, cantonal tax, and also for general social insurances.

*Gini_pre*_*i*_ is computed according to the following formula:

$$y\_\mathrm{pr}e_{h}=\sum_{k=1}^{N}y_{\mathrm{hk}}-\sum_{s=1}^{m}\left(1-\mu_{s}\right)sc_{\mathrm{hs}}-\sum_{v=1}^{p}\left(1-\tau_{v}\right)t_{\mathrm{hv}}$$

where:

• $\sum_{k=1}^{N}y_{\mathrm{hk}}$ is the gross income for the household *h*, which, according to the definition given in [3], consists of the sum of all income earned from work and self-employment, interest, house rental, social insurance benefits, revenues from other insurances, and other indemnities.

• $\sum_{s=1}^{m}\left(1-\mu_{s}\right)*sc_{\mathrm{hs}}$ is the part of the general social insurance paid by the household *h* and not directed towards financing health care. In particular, *μ*_*s*_ is the share of health care services funded out of the budget of the social insurances. In 2005, *μ*_*s*_ was 3.3 percent for the pension and the disability insurance, and 24.5 percent for the accident insurance.

• $\sum_{v=1}^{p}\left(1-\tau_{v}\right)*t_{\mathrm{hv}}$ are the taxes (federal, cantonal and municipal) paid by the household *h* and not directed towards financing the healthcare system. As in [3], federal indirect taxes were proxied with VAT, which accounts for two-thirds of total indirect taxes. VAT has been computed from the data on consumptions that household declared in the survey.

• As in the case of social contribution, *τ*_*v*_ is the part used to finance the health care system for each type of tax (*v*), so 1-*τ*_*v*_ is the residual part that is not used within the health sector.

• To give an idea of the dimension of these coefficients, in 2005 *τ*_*v*_ was 5.4 percent for federal tax, 28.5 percent (on average) for cantonal tax and 4.3 percent (on average) for communal tax.

Disposable income before health-care financing has been corrected using an equivalence scale in order to make households with a different number of members comparable^e^.

Moreover, as different years are pooled together, the values have been deflated through the consumer price index (CPI) for Switzerland.

Kakwani is a useful index for providing information about the shift from proportionality. However, it is an extremely summary measure, and its information can be sometimes misleading if the distributions underlying the Kakwani index are not considered alike. When curves cross, a value of Kakwani equal to zero could be the result of a concentration curve that is progressive for half of the population and regressive for the other half. To control for this, we look also at the concentration curves and the relative dominance test, that aims to define statistically whether one curve dominates another. The null hypothesis indicates that there is no significant difference between the two concentration curves considered. The choice criterion used here is the *multiple comparison approach*. In the comparison between the two curves, the null is rejected if there is at least one significant difference between them in one direction and no significant difference in the other direction. The ordinates are compared in 19 different quintiles, as suggested by O’Donnell et al. [26].

## Results

Table 3 presents the results of the Kakwani index over time for the whole Switzerland and for the seven macro-regions.

**Table 3:** Kakwani index over time

	**1998**	**2000**	**2001**	**2002**	**2003**	**2004**	**2005**
***Macro-region 1***	-0.136** [1495]	-0.091** [590]	-0.069** [586]	-0.065** [603]	-0.099** [528]	-0.090** [505]	-0.074** [502]
***Macro-region 2***	-0.106** [2173]	-0.081** [925]	-0.081** [907]	-0.109** [840]	-0.105** [788]	-0.085** [780]	-0.109** [751]
***Macro-region 3***	-0.103** [1287]	-0.099** [504]	-0.044* [516]	-0.062** [529]	-0.065** [482]	-0.076** [441]	-0.084** [400]
***Macro-region 4***	-0.130** [1659]	-0.138** [638]	-0.092** [612]	-0.094** [640]	-0.104** [622]	-0.111** [562]	-0.112** [531]
***Macro-region 5***	-0.130** [1183]	-0.116** [433]	-0.125** [478]	-0.119** [469]	-0.084** [438]	-0.137** [420]	-0.147** [417]
***Macro-region 6***	-0.136** [806]	-0.135** [329]	-0.080* [300]	-0.089** [313]	-0.108** [289]	-0.125** [287]	-0.075** [245]
***Macro-region 7***	-0.087** [692]	-0.074** [223]	-0.078** [341]	-0.034 [332]	-0.027** [328]	-0.083** [275]	-0.016** [241]
***CH***	-0.130** [9295]	-0.110** [3642]	-0.084** [3740]	-0.094** [3726]	-0.098** [3475]	-0.104** [3270]	-0.101* [3087]

Significance level: *p < 0.05, **p < 0.01.

The values in brackets show the sample size.

The most notable result is that the total value of the Kakwani index is always negative, which means that the Swiss health-care system financing remains regressive, even after the major reform of 1996; this result is in line with the previous literature. The other notable point is that the results do not seem to vary widely in the years considered, neither for all of Switzerland nor for any of the macro-regions. The most regressive year appears to be 1998, although the data provides no evidence of any clear temporal trend.

Macro-region 7 (Ticino) seems to reach the best value in terms of equity, for all the years considered, since the Kakwani is not statistically different from zero in 2002, 2003 and 2005. Macro-region 4 (Zurich) is in line with the Swiss average, and macro-region 3 (Basel City, Basel Land, and Argovia) is, after Ticino, the least regressive group of cantons.

The weighted Kakwani index for the SHE in Switzerland ranged from -0.084, reached in 2001, and -0.13 in 1998^f^.

Table 4 presents the Kakwani indexes for the Swiss cantons and helps shed light on how fiscal federalism allows cantons to choose their preferred financing policy.

**Table 4:** Kakwani index across cantons

	**Number of observations**	**Federal taxes**	**Cantonal and municipal taxes**	**General social insurances**	**Mandatory health insurance (net premiums)**	**Weighted Kakwani index**
ZH	*5264*	0.0088	0.0389	0.0074	-0.2548**	-0.1085**
BE	*4191*	-0.0052	-0.0121	0.0330**	-0.20806**	-0.0947**
LU	*1353*	-0.0069	0.0181	0.0560**	-0.2298**	-0.0936**
SZ	*450*	-0.0578	-0.0417	0.0025	-0.2508**	-0.1344**
OW	*129*	0.0387	0.0744	0.0999**	-0.2323**	-0.0567**
NW	*158*	0.0236	0.1256	0.0594	-0.2738**	-0.0729**
GL	*138*	0.0063	0.0135	0.0366	-0.2304**	-0.0913**
ZG	*375*	0.1093	0.0540	-0.0230	-0.2603**	-0.0871**
FR	*916*	-0.0159	-0.0121	0.0246	-0.1863**	-0.0874**
SO	*1013*	-0.0311	0.0473**	0.0536**	-0.2109**	-0.0868**
BS	*782*	0.0485	0.0835	-0.0001	-0.2149**	-0.0582**
BL	*1066*	0.0557*	0.0933**	-0.0042	-0.2191**	-0.0774**
SH	*295*	-0.0963	-0.1310	-0.0059	-0.2239**	-0.1535**
SG	*1648*	0.0011	0.0407	0.0369*	-0.2571**	-0.1066**
GR	*723*	-0.0481	-0.0327	0.0231	-0.2605**	-0.1285**
AG	*2311*	0.0200	0.0308	0.0147	-0.2250**	-0.1065**
TG	*806*	0.0251	-0.0372	0.0406*	-0.2281**	-0.1131**
TI	*2432*	0.0122	0.0850**	0.0310*	-0.1800**	-0.0592**
VD	*2531*	-0.0148	0.0131	-0.0099	-0.2050**	-0.1004**
VS	*1083*	-0.0464	-0.0435	-0.0006	-0.2123**	-0.1114**
NE	*776*	-0.0526	0.0306	-0.0035	-0.2009**	-0.0904**
GE	*1195*	0.0277	0.0976	-0.0673**	-0.2482**	-0.0643*
JU	*268*	-0.0076	0.0799	0.0549	-0.1809**	-0.0566**
**CH**	***30232***	**0.0074**	**0.0153**	0.0090*	-0.2271**	-0.0999**

Significance level: *p < 0.05, **p < 0.01.

It is evident from the table that there are important differences in the regressivity of the financing system among cantons.

The Kakwani index for *federal taxes* is not statistically different from zero for all the cantons apart from canton Basel Land (BL), which has a slightly progressive value. This means that federal taxes are concentrated quite proportionally among the population. This result could be easily explained if we think that the tax amount considered here is the sum of a progressive federal direct tax (based on income) and an indirect tax (VAT) based on consumption that is normally regressive. Canton Basel Land is the only one in which the effect of the progressive direct tax more than offsets the effect of the regressive VAT.

The Kakwani indexes for the *cantonal and municipal taxes* are more difficult to explain. While we would expect a highly progressive value, most of them are not statistically different from zero. Only Solothurn (SO), Ticino (TI), and Basel Land (BL) present a slightly positive value of the Kakwani index, which means a slightly progressive tax. These values are smaller than the results expected, but there are at least two reasons that could explain them.

First of all, this financing source includes both cantonal and municipal tax. Very rich households may choose to live in jurisdictions that have a lower local (municipal) taxation. Therefore, a richer household may have to pay the same cantonal tax, but a different (lower) municipal tax than a poorer household that lives in a municipality with higher taxation. This could bias the progressivity results in favor of a score that cannot reject the hypothesis of proportionality.

A second reason could be the fact that there is a mismatching between household income that refers to the year of the interview and household taxes that are paid on income earned the year prior to the interview. There could be situations in which a person has reduced his or her revenues (perhaps because they have temporarily lost their job or have retired), but reports to pay high taxes because she/he earned a “regular” income in the previous year. This could worsen the situation of regressivity because the Kakwani index is computed as if tax paid and income earned referred always to the same year^g^.

As expected, the Kakwani indexes for the *general social insurance* are not statistically different from zero. Since the tax rate is the same for every wage earned, regardless of its level, we expected a Kakwani value near to zero. Nevertheless, there are some exceptions. Four cantons (Bern, Lucerne, Obwald, and Solothurn) present a progressive value, while canton Geneva is the only one that has a regressive index for this financing source. This could be because the share of capital income over the total income in Geneva is higher than in the other cantons (and, consequently the wage income share is lower): the fact that the payment for the social insurance is only based on wage income could induce a regressive effect in the results.

The most interesting results are those for the *mandatory health insurance*. They are all significant for each canton and range from a value of -0.18 for canton Ticino to -0.27 for canton Nidwald.

It is quite notable that the values can be so different, even in neighboring cantons: the value of the index in canton Grisons (-0.26) is 0.08 less than that of its neighbor, Ticino (-0.18). Based on the Welch’s t-test, we can show that this difference is statistically significant at the 95% level. This outcome probably hides a different choice in the subsidy policy that cantonal authorities are free to set-up. Previous studies (see [21,23]) have shown that on average the same family (e.g. parents with two children and a revenue of 70’000 CHF) has to devote 4.4% of disposable income if it lives in Obwald, but up to 16.4% if the canton of residence is Vaud. This heterogeneity jointly reflects the premium level and the generosity of the subsidy policy. In general cantons with a very high level of premiums prefer to distribute generous subsidies to a limited group of citizens, whereas those with lower premiums levels choose to reach more people by distributing lower subsidy amounts [4]. There is one clear outlier-canton which distributes higher subsidies than the average to an above-average share of the population, namely Ticino (Crivelli et al. [27] show that the subsidy policy in Ticino is 34.5 percent more generous in terms of CHF spent per capita than the Swiss average). This might explain why in Ticino the net premiums are less regressive than elsewhere. On the other hand Grisons distributes subsidies that are below the Swiss average and support only a limited number of people. This strategy seems to be in line with the computed Kakwani for MHI, which is in the case of Grisons the most regressive one.

Finally, the results on the *total* Kakwani indices are all statistically significant^h^ and all negative.

The cantons that appear to be the most regressive ones are canton Schaffhausen and Schwyz, with a Kakwani equal to -0.15 and -0.13, respectively. The cantons that reach the best results in terms of equity are Jura, Obwald and Ticino, all of which have a Kakwani value of around -0.05.

It is worth noting that the difference between the least (Jura) and the most regressive canton (Schaffhausen) is considerable (approximately 0.10) and significant at 99% level (according to the Welch’s t-test); it is the same as the difference between the US (-0.13) and Sweden (-0.015) from the Wagstaff et al.’s (1999) study, countries that have two completely different health-care financing systems. Although Shaffausen and the Jura have greater absolute levels of regressivity than the US and Sweden respectively.

We now report the concentration curves and the dominance tests results.

A general summary comment, based only on the visual inspection of the concentration curves, would suggest that their order follows a similar pattern for all the cantons. Firstly, starting from the 45-degree line and going down, the net premium curve is always the first one, very close to the 45-degree line and not crossing with the others. Secondly, the social contribution curve is generally the next one, always very near to the Lorenz curve, which indicates proportionality. Thirdly, the federal tax line is also quite near to the Lorenz curve and, finally, the cantonal tax curve is always the most distant and lies under the Lorenz, indicating progressivity. Nevertheless, the two curves for taxes and the one relative to the social insurance often cross among them and with the Lorenz, which indicates an alternation between regressivity among the poorer and progressivity among the richer.

We have chosen to show the results only for canton Geneva, which is among the least regressive.

**Figure 1:**
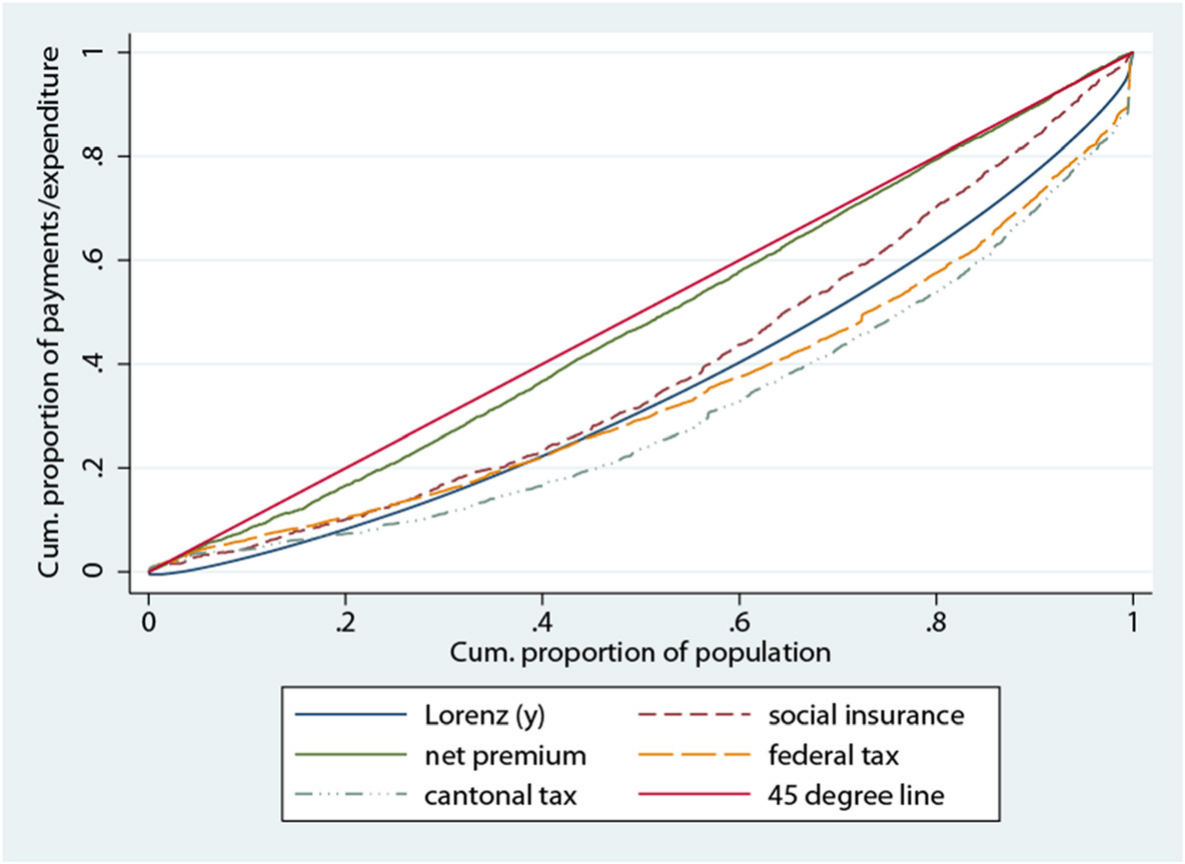
Concentration curves for each financing source, canton Geneva.

Figure 1 represents the concentration curves of each financing source. The concentration curve for the net premium is very close to the 45-degree line, indicating quite strong regressivity, and it dominates the Lorenz curve. In Table 5 we read that the poorest 30 percent of the population receives about the 15 percent of the total income and pays 26 percent of the net premium.

**Table 5:** Cumulative shares of income and health payments by income decile, canton Geneva

**Quantile**	**Cum. share of income**	**Cum. share of federal taxes**	**Cum. share of cantonal and municipal taxes**	**Cum. share of general social insurances**	**Cum. share of mandatory health insurance (net premiums)**
*Q10*	2.78%	6.26%**	4.32%	4.57%	8.08%**
*Q20*	8.24%	10.55%*	7.40%	10.06%	16.62%**
*Q30*	14.75%	15.61%	11.21%*	16.87%	26.16%**
*Q40*	22.35%	22.04%	16.60%**	23.15%	36.71%**
*Q50*	30.82%	29.29%	23.69%**	31.75%	47.10%**
*Q60*	40.31%	37.41%	32.80%**	43.70%*	57.73%**
*Q70*	50.92%	46.08%*	42.95%**	55.78%**	68.60%**
*Q80*	62.85%	57.53%*	53.94%**	70.27%**	79.40%**
*Q90*	76.98%	71.94%	69.61%*	83.72%**	89.47%**

Significance level: *p < 0.05, **p < 0.01. The significance level indicates whether the percentage share of financing.

source is significantly different from the *income_pre*.

The tests of dominance (not shown in the table) find evidence of dominance of the social contributions curve on the Lorenz curve and dominance of the Lorenz curve on the cantonal tax curve. These two last results tell us two important things. Firstly, the dominance for the social contribution curve confirms the result of the Kakwani index: the social contributions are regressive in Geneva, while the situation is different for the cantonal tax. This financing source seems to be progressive, but only starting from the second decile of the ranked population (i.e., for the richest 80 percent). For the poorest 20 percent of the ranked population, we cannot reject the hypothesis of proportionality (as the difference between the two curves is not statistically significant).

## Discussion: lessons for a federal state

The most relevant evidence is that the regressivity of the MHI implies that the subsidy policy adopted by the state does not succeed in making the financing of this source progressive or, at least, proportional. Considering that the subsidy is the most important tool that cantons have to mitigate the regressive nature of the community-rated premiums, this is a very strong result for policy makers.

However, the value of Kakwani here includes something more than the effects of the earmarked subsidies chosen by the cantons. Also, the individual choices (premium and deductible) allowed by the competitive setting of the Swiss health insurance have a significant impact on equity. The Swiss system encourages competition between insurance companies, which plays out in the level of the premium and in the quality of service; twice per year, people have the option to switch to another insurance company (with lower premiums, e.g.). Moreover, citizens are allowed to obtain a discount on the monthly premiums by choosing a higher deductible; by assuming some of the financial consequences of getting sick themselves, they can pay a smaller monthly premium^i^. Finally, people who choose to be part of a managed care plan can also get a discount on premiums. These choice options are not constrained to a certain level of income, but there is evidence that not all citizens can manage the available information to make a rational choice in terms of switching [21] or of selecting the appropriate type of contracts. Accordingly, we cannot exclude the existence of a social gradient in the ability to manage information. In this case, the burden of solidarity with the sick implied by community rating may be partially shifted back from the good risk to the bad risk as well as from the better off to the worse off, with negative consequences on the equity level.

Similarly, the Kakwani index of taxation includes also an indirect (or endogenous) effect due to individual choices. For example, very rich people may decide to live in a canton (or municipality) with a less progressive taxation to pay less taxes and this may also have an impact on the regressivity level of the canton. If this effect increases or decreases the regressivity depends on the distribution of income among the population.

Another consideration needs to be specified. The Kakwani index considers only the relative financing burden of households with respect to others, not the absolute burden of the health financing. The poor in a canton with a slightly regressive financing may have to pay absolutely more for health care than people with the same income in a canton with highly regressive financing.

In other words, the Kakwani index is appropriate to measure the level of vertical inequity, but it is not able to capture the extent of horizontal inequity.

To have an idea of the dimension of horizontal inequity, we computed the *incidence* of health expenditure on the total income for different income classes. We found that there is a similar general trend for all cantons; specifically, the incidence for the lower-income classes is heavy and decreases for the richer classes.

**Figure 2:**
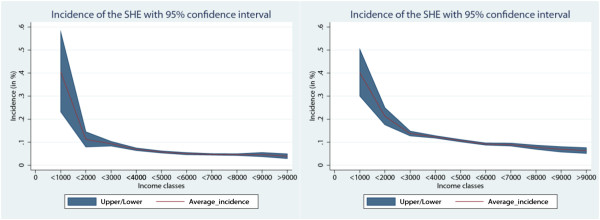
Average incidence of the socialized health expenditure on income, Zug (left) and Geneva (right).

To exemplify this, the graphic representations for the incidence of the financing burden for the cantons of Zug and Geneva are reported below (Figure 2).

The red line represents the average incidence for each of the income classes considered, while the blue-shadow area indicates the confidence intervals. Interestingly, the two red lines have a similar shape, although the line for Geneva is shifted upwards with respect to the other.

With an exception for the first income class, the level of incidence is quite different: the share of income that people in Geneva pay is almost double that of citizens earning an equivalent income in Zug. This means that people in Geneva (one of the least inequitable cantons) pay more, not only in absolute value, but also as a percentage on their income than in Zug (one of the most inequitable cantons in terms of the Kakwani analysis).

The reason for such a result can easily be found by simply looking again at the absolute level of expenditure (Table 2): Geneva registers one of the highest levels (6578 CHF per capita), while Zug has one of the lowest ones (3885 CHF per capita).

This example clearly shows that the burden of health-care financing may be equally distributed, but may be too heavy to be affordable for lower- and middle-income people. To have a clear understanding of the equity issue, the importance of the absolute expenditure levels cannot be neglected.

The fact that people of the same income class show a different incidence level across cantons suggests the presence of horizontal inequity. We would like to highlight a final remark upon this issue. The Swiss federalism allows some form of autonomy to cantons, especially in deciding the subsidy policy. Therefore, subsidies are an important tool in reducing the vertical inequity within a canton, but, on the other hand, being administered differently in each canton, they may also lead to further horizontal inequity, i.e. to more inequity across cantons.

Finally, we cannot disregard that one possible criticism with respect to the equity issue is the potential trade-off between equity and efficiency, according to what Okun theorized in 1975 [28]. To check for this, we considered the efficiency results found in a study by Widmer and Zweifel [29] in which they performed a data envelopment analysis for the Swiss cantons for six categories of public goods, including health, using data relative to the years 2000–2004. We ranked the cantons first according to our equity results and then according to the efficiency results found by the two authors. We compared the two ranking lists through a Spearman test and a Kendall test of rank correlation, but the results do not reject the null hypothesis of independence. This leads to the conclusion that there is no direct link between equity and efficiency in this particular setting.

## Conclusions

This study represents the first attempt to investigate the impact of federalism on the financing of a universal health insurance system in terms of equity.

We have analyzed the financing of the Swiss health-care system from an equity point of view, with particular attention to the differences across cantons. We used the Kakwani index for each financing source to see how each of them shifts from proportionality. The dominance test for the concentration curves has also been performed to exploit more the available information.

The general results suggest that Swiss health-care system financing is regressive in all cantons, although there are huge differences among them. The reason for this lies in the federal setting, which allows cantons to have some freedom in certain areas, such as how to design subsidy policies for lower-income people, the choice of tax rates, and the choice of how much of the total expenditure has to be financed through taxation and how much through mandatory health insurance. These factors, along with the characteristics of the Swiss health-care system based on competition among insurance companies and on the supply of premium discounts for people opting for higher deductibles and managed care contracts, bring different levels of inequity. The results highlight the fact that the level of regressivity of mandatory health insurance premiums (net of subsidies) varies significantly across cantons, whereas for federal taxes and general social insurance the hypothesis of proportionality cannot be rejected in the majority of cantons. Finally, the cantonal tax is progressive only in three cantons.

The main limitations of our paper are due to the dataset on which we relied; we had to aggregate data of seven different years to get robust estimates at the cantonal level. Moreover, we had to focus on SHE because the information on out-of-pocket expenditure was not reliable for six out of seven years. Since 2008, a new legislation has been enforced in Switzerland that assigns to cantonal authorities even more leeway regarding the subsidy policy (the matching grants transfer has been replaced by a lump-sum payment). It is therefore necessary to monitor the situation in more recent years in order to figure out how inequity developed in Swiss cantons after this reform.

The results contained in this work shed light on some aspects that should be considered in other federal states that are planning to use the regulation of private health insurance and premium subsidies to ensure universal coverage. Although the combination of community rating and premium subsidies might be in theory the best solution in terms of (vertical) equity [30], in the daily practice of federal states the empirical evidence can point to a different outcome. In fact, tax competition and a “race to the bottom” in social policy (in order to avoid the outflow of good taxpayers and the inflow of people looking for social aid) may jointly lead to a lower level of vertical equity than a sovereign state would choose if the design of social policy would occur at the national level^l^.

## Endnotes

^a^E.g. until 2011 cantons had significant leeway in allocating the public financing to private hospitals. This has been changed by the new regulation of hospital financing that started in 2012.

^b^The available data does not provide any information about the direct federal health expenditure given to each canton or the amount of health expenditure from the general social insurance spent in each canton. To compute these two values, we took the total amount of expenditure and simply imputed the same average spending for each Swiss inhabitant, regardless of the canton in which she/he lives. In the expenditure of the confederation and the cantons, we also accounted for the amount of the subsidies distributed.

^c^Macro-region 1 includes Vaud, Valais, and Geneva; macro-region 2 includes Bern, Fribourg, Solothurn, Neuchâtel, and Jura; macro-region 3 includes Basel City, Basel Land, and Argovia; macro-region 4 includes Zurich; macro-region 5 includes Glarus, Schaffhausen, Appenzell O. Rh., Appenzell I.. Rh., St. Gall, Grisons, and Thurgovia; macro-region 6 includes Lucerne, Uri, Schwyz, Nidwald, Obwald, and Zug; macro-region 7 includes Ticino.

^d^Canton Uri, canton Appenzell I. Rhodes, and canton Appenzell O. Rhodes have not been considered because the small number of observations in the dataset did not allow for any robust computation.

^e^The scale chosen is the OECD modified equivalence scale, which assigns a value of 1 to the household head, of 0.5 to each additional adult member and of 0.3 to each child no older than 13.

^f^As explained in section 4, data for 1998 was collected in a different manner that makes it possible to compute the Kakwani index for the total health expenditure, including the out-of-pocket payment and the complementary insurance. We computed this for all of Switzerland and found that the equity results are even worse (Kakwani -0.14).

^g^There is another factor to consider: the tax reform. From 1990 until 2003 cantons, in turn, had to adapt their system of taxation to a new one. This passage provoked an asymmetry between the tax paid in one year and the income earned in the same year that may have brought some additional asymmetries also in our computations.

^h^The standard error for the total Kakwani index has been obtained summing the standard errors for the single components (*i*) according to the formula: $\mathrm{se}=\sqrt{\sum_{i}\left(w_{i}se_{i}^{2}\right)}+2\sum_{\mathrm{ij}}\left(w_{i}w_{j}\mathrm{co}v_{\mathrm{ij}}\right)\;\forall i\neq j$.

^i^Financially constrained households may ‘underinsure’ by choosing higher deductibles or switching to a more restrictive insurance plan (managed care). Hence they may incur higher out of pocket payments, which are not measured in the study and which would underestimate the inequity level.

^l^In 1991, through the HIA draft bill, the Swiss federal government suggested to fix the maximum incidence level for health insurance premiums at 8% of the taxable income. However, this ‘social target’ was not anchored in the law and cantons were entrusted with large autonomy in the design of their subsidy scheme. In actuality, in several cantons a large part of the population pays for premiums a share that significantly exceeds this threshold [21,23].

## Competing interests

The authors declare that they have no competing interests.

## Authors’ contributions

PS surveyed the literature, prepared the dataset and carried out all the computations. PS and LC drafted the manuscript together. Both authors read and approved the final manuscript.
